# Atypical Presentation of an Odontogenic Brain Abscess Due to Eggerthella lenta and Bilophila wadsworthia Coinfection

**DOI:** 10.7759/cureus.104945

**Published:** 2026-03-09

**Authors:** Man Fung Cheng, Ernest Dodoo

**Affiliations:** 1 Neurosurgery, Princess Margaret Hospital, Lai Chi Kok, HKG

**Keywords:** anaerobe, antibiotic therapy, bilophila wadsworthia, brain abscess, culture, eggerthella lenta, microbiological culture, neuroradiology, odontogenic, periodontitis

## Abstract

Odontogenic brain abscesses are uncommon. *Eggerthella lenta* and *Bilophila wadsworthia* are anaerobes that are normal components of the human intestinal microbiota. Both can also be found in the oral cavity. We report the case of an 81-year-old man with a brain abscess caused by coinfection of *E. lenta* and *B. wadsworthia* associated with periodontitis. The patient underwent a right frontal craniotomy with excision of the abscess. Ceftriaxone and metronidazole were then started, along with levetiracetam. Ceftriaxone and metronidazole were maintained for four weeks, and the antibiotic treatment was switched to oral cephalexin and metronidazole for an additional two weeks. Immune senescence and bacterial translocation play an important role in pathogenesis. Early detection, surgical excision of the abscess, and antibiotic therapy resulted in a favorable clinical outcome.

## Introduction

Brain abscess is a focal infection characterized by circumscribed cerebritis in the early phase, followed by the subsequent development of a pseudo-capsule surrounding purulent exudate [1]. The incidence of brain abscess is 0.3-0.9 per 100,000 inhabitants in developed countries, with mortality ranging from 17% to 37% [2]. New-onset epilepsy occurs in 32% of 30-day survivors [3]. The most common etiology (40-50%) is the spread of contiguous infection from sinusitis, otitis media, or oral infection [4,5]. Hematogenous spread from distant sources accounts for 30% of brain abscess cases [6]. Traumatic brain injury or neurosurgery accounts for 10% of cases, and in 10-20% of cases, no etiology is clearly identified [7]. Odontogenic infection is relatively uncommon [8], and the number of reported brain abscess cases due to dental pathogens is limited [9,10]. We present a case in which bacteria of odontogenic origin were identified in the purulent material of a patient with a brain abscess. Severe periodontitis was also recognized.

## Case presentation

An 81-year-old male patient with diverticulosis and hypertension was admitted to the emergency department because of sudden-onset left facial twitching and weakness. He was noted to have mild slurring of speech. He denied fever, nausea, vomiting, or headache. No abdominal pain or rectal bleeding was reported. Physical examination revealed a temperature of 36.6°C, blood pressure of 150/64 mmHg, and pulse of 81 beats per minute. His Glasgow Coma Scale was 15/15 [11]. Limb power was full, and the facial twitching had subsided. No facial weakness was detected.

**Figure 1:**
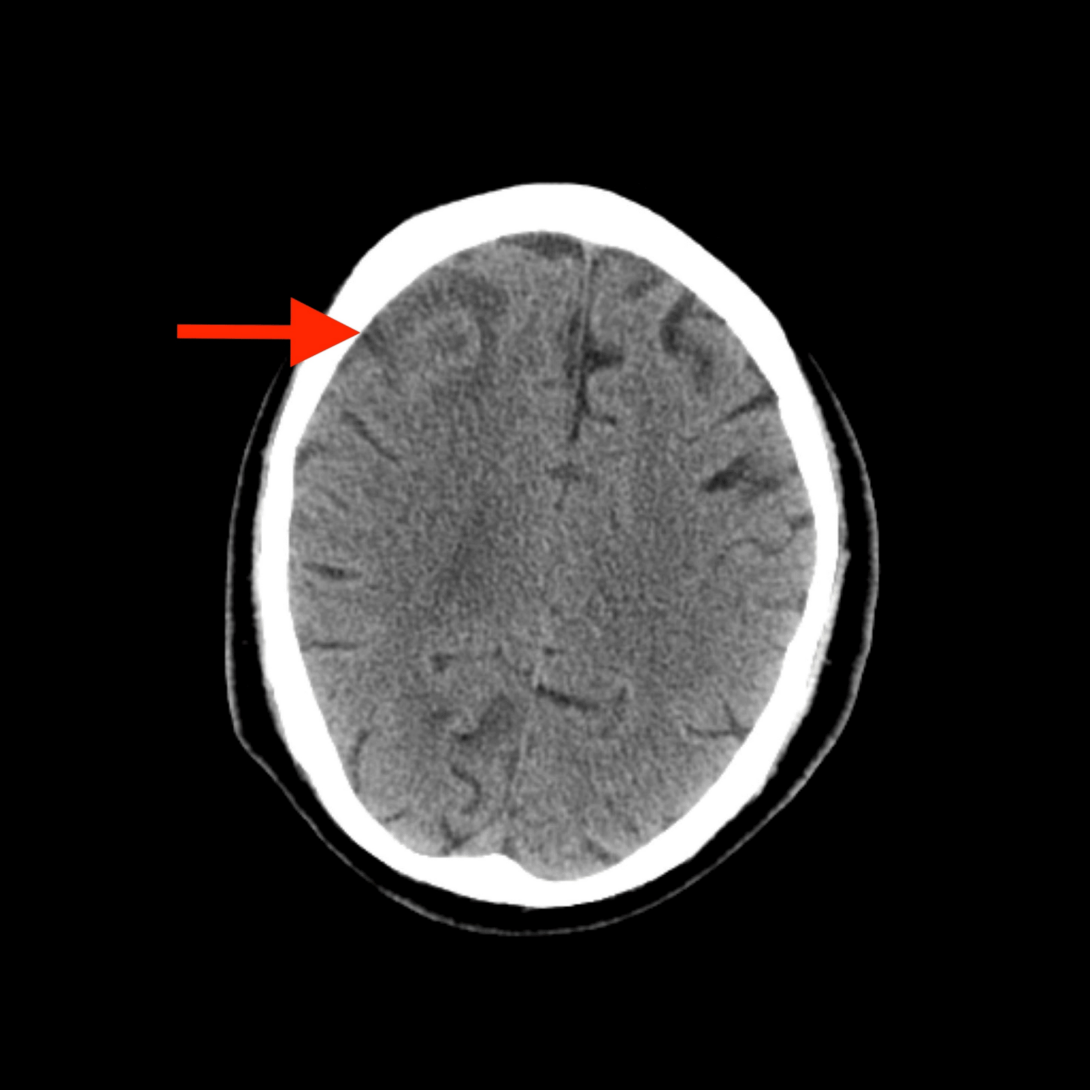
Non-contrast CT of the brain showing a right frontal mass with perilesional edema Axial view demonstrating a mass (red arrow) in the right frontal lobe, exhibiting surrounding vasogenic edema.

**Figure 2:**
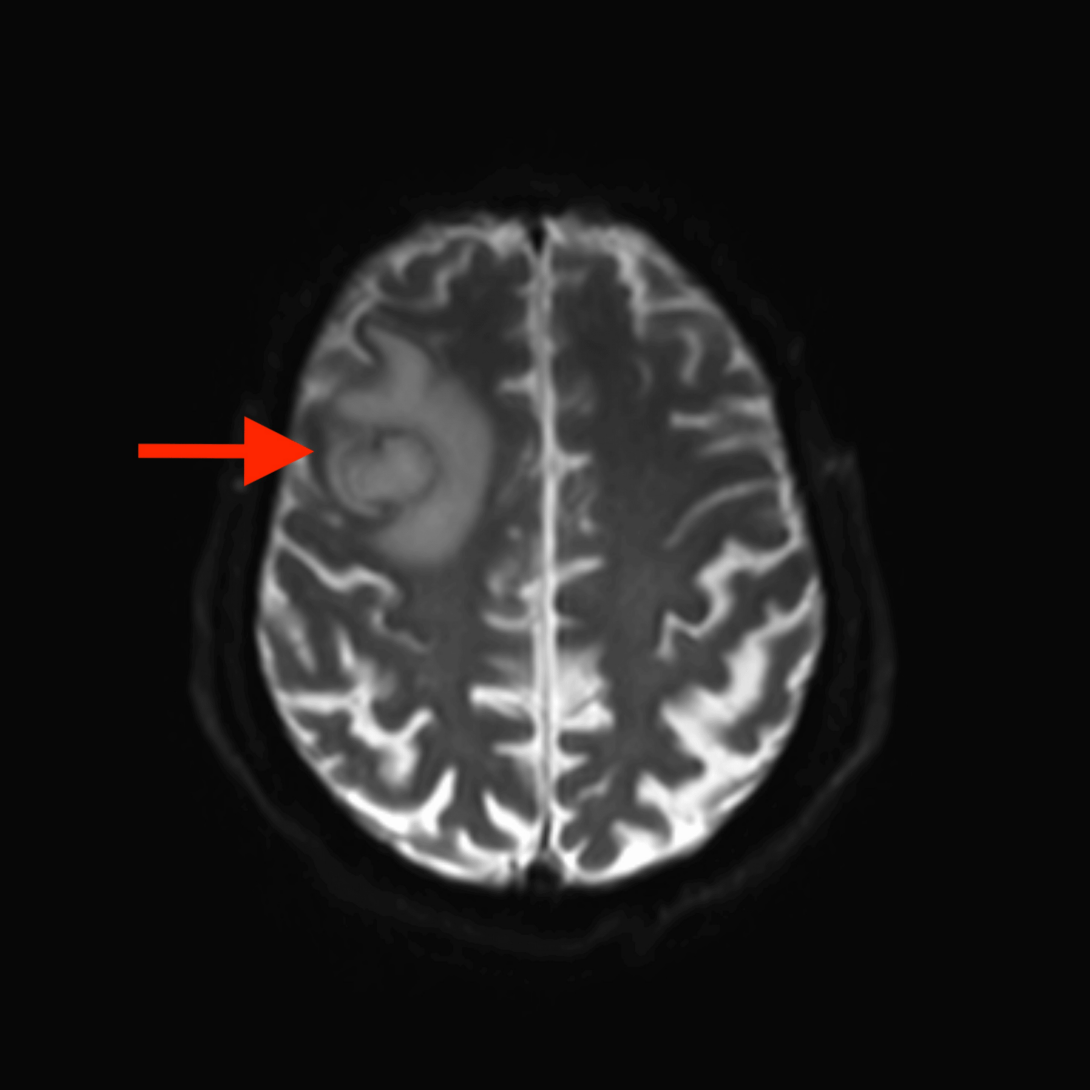
MRI of the brain showing a right frontal lesion with restricted diffusion

CT of the brain showed a right frontal lesion with peripheral edema (Figure 1). Cerebral neoplasm was suspected, with a differential diagnosis including a brain abscess. Tumor markers, such as carcinoembryonic antigen, alpha-fetoprotein, and prostate-specific antigen, were negative. HIV serology was negative, and HbA1c was 6.0%. MRI of the brain showed a ring-enhancing lesion with internal restricted diffusion on diffusion-weighted imaging (DWI) (Figure 2).

PET/CT scan showed no positive uptake in the trunk, abdomen, or pelvis. Intravenous dexamethasone 4 mg every six hours and phenytoin were started. The patient was transferred to our unit. Upon arrival, erythrocyte sedimentation rate was 19 mm/hour, C-reactive protein was 5.7 mg/L, and white blood cell count was 15 × 10⁹/L.

The patient underwent a right frontal craniotomy with total excision of the lesion. Aspiration of the lesion yielded pus-like content. Abscess specimens were cultured on blood agar, chocolate agar, and MacConkey agar, followed by incubation at 35°C for two days. Specimens were also inoculated into BD BACTEC™ Plus Aerobic/F and BD BACTEC™ Lytic Anaerobic/F culture vials and incubated using the BACTEC FX instrument (BD, Franklin Lakes, NJ, USA) for up to seven days. When the BACTEC FX system indicated positive growth, the cultures were subcultured onto blood agar, chocolate agar, MacConkey agar, and anaerobic agar for further incubation for an additional two days. The resulting isolates were identified using MALDI-TOF MS (IVD, Bruker Daltonics, Billerica, MA, USA).

A mixed culture of *Eggerthella lenta *and *Bilophila wadsworthia *was identified. Both species were considered clinically significant. Molecular methods, such as 16S rRNA sequencing and metagenomic next-generation sequencing (mNGS), were not available as routine diagnostic services in our laboratory. The two cultured bacteria were sensitive to metronidazole. Pathology of the excision specimen confirmed an abscess wall.

**Figure 3:**
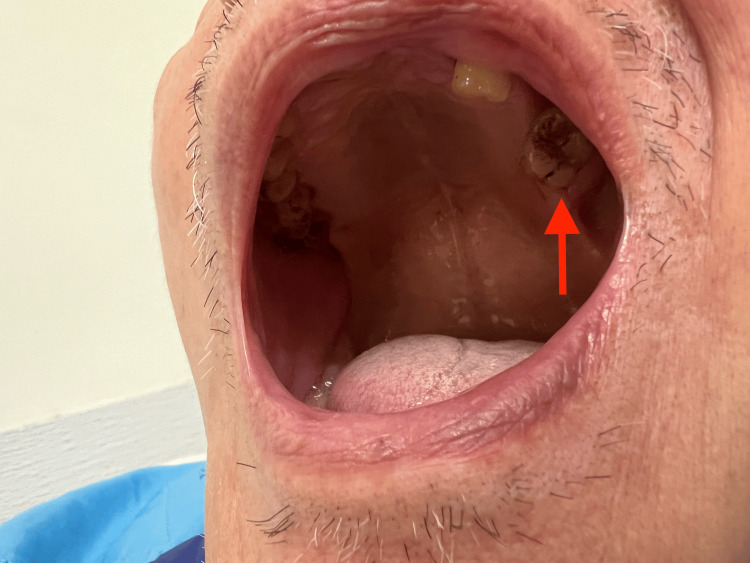
Poor oral hygiene with dental plaque (red arrow) and loss of teeth The clinical photo was taken after admission to our unit. Dental plaques on the left upper molars and missing teeth were observed.

**Figure 4:**
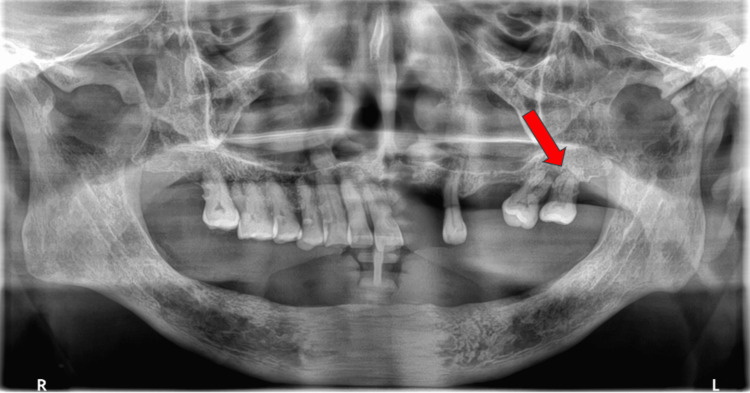
OPG showing left upper third molar with apical bone loss (red arrow) Apical bone loss, a feature of severe periodontitis, involving tooth 28, was observed along with loss of adjacent teeth. OPG, orthopantomogram

Dental examination revealed poor oral hygiene with plaque and loss of teeth. Miller II hypermobility of the left upper molar tooth was detected (Figure 3) [12]. An orthopantomogram (OPG) showed apical bone loss at tooth 28 (Figure 4).

OPG showed apical bone loss at tooth 28 (Figure 3). Extraction of tooth 28 was performed by the dental team at our institution. Transthoracic echocardiogram showed no patent foramen ovale or valvular vegetation. Contrast-enhanced CT of the abdomen and pelvis demonstrated diverticulosis without evidence of diverticulitis or intra-abdominal collection.

Intravenous metronidazole 500 mg every eight hours and ceftriaxone 2 g every 12 hours were started. Phenytoin was replaced with levetiracetam due to a mildly elevated alanine transaminase level (53 IU/L). The patient continued on ceftriaxone and metronidazole for four weeks. Oral cephalexin and metronidazole, as per our institutional practice, were then commenced for an additional two weeks. The patient remained afebrile and was discharged neurologically intact, with a modified Barthel Index score of 89/100 [13].

**Figure 5:**
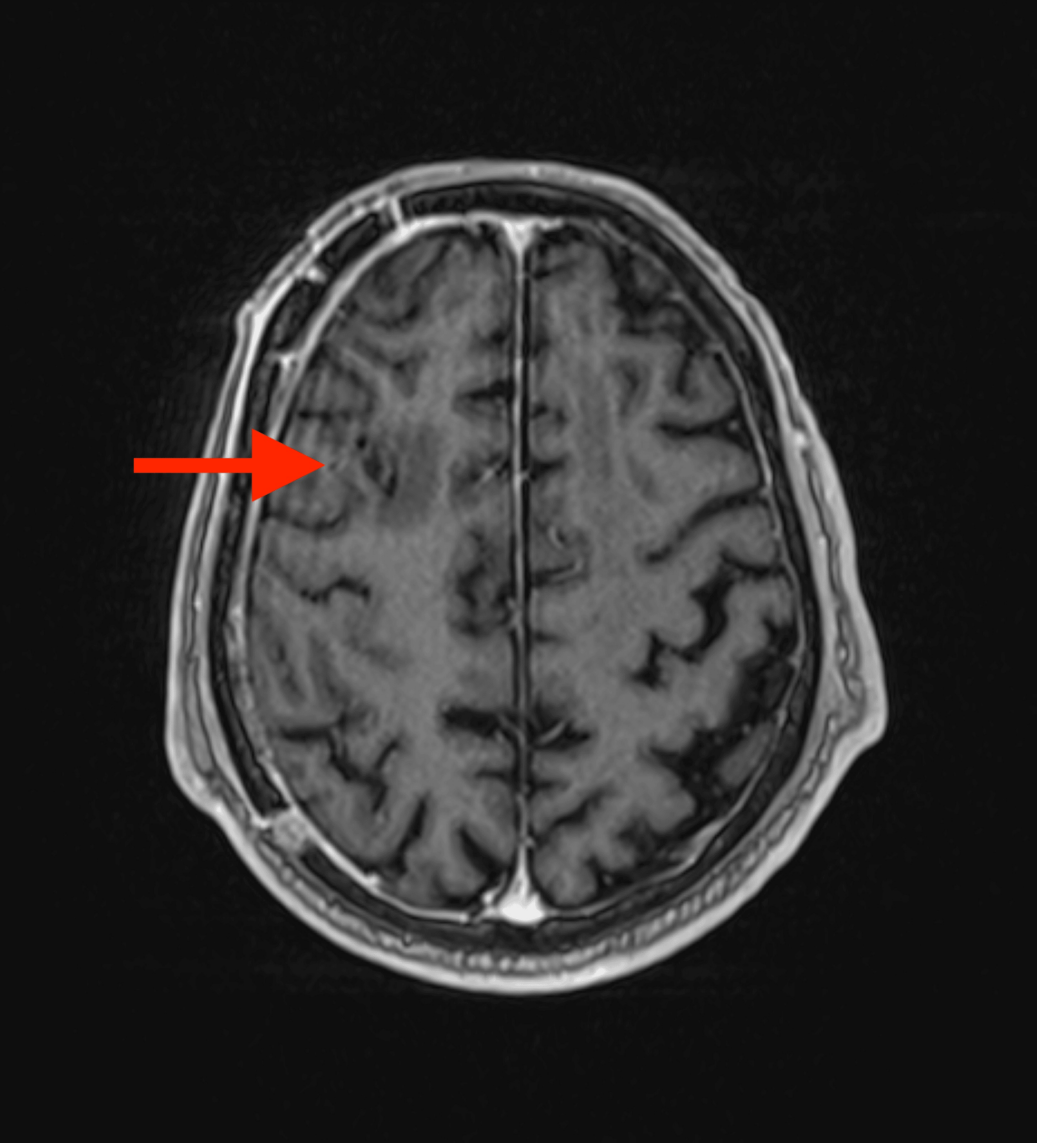
MRI of the brain showing resolved right frontal abscess, 10 weeks after surgery

Subsequent MRI revealed resolution of the abscess (Figure 5).

## Discussion

The epidemiology of brain abscesses varies considerably depending on socioeconomic factors and the health status of populations [14]. The triad of headache, fever, and focal neurological deficit is observed in only 20% of cases [15]. The presenting symptoms in our case were a partial seizure and limb weakness. Non-specific or stroke-like symptoms may lead to delays in diagnosis and treatment [16,17]. High awareness and early utilization of imaging techniques facilitated timely diagnosis. The mortality of anaerobic brain abscesses is reported at 6.3%, with a male predominance [18].

The incidence of brain abscesses caused by anaerobes has been underestimated due to the growth conditions required for isolation, challenges with identification, and the fastidious nature of anaerobes [19]. The most common cause of anaerobic brain abscesses is contiguous infection, especially from an otogenic focus, followed by prior neurosurgical procedures and hematogenous spread [18]. Odontogenic brain abscesses are relatively infrequent [20].

Contrast-enhanced CT has lower sensitivity and specificity than MRI [21]. The sensitivity and specificity of MRI in detecting brain abscesses are 92% and 91%, respectively [22,23], with corresponding positive and negative predictive values of 88% and 90%. DWI has been confirmed as a reliable tool to distinguish brain abscesses from malignant ring-enhancing brain lesions [24]. Restricted diffusion within a ring-enhancing mass is characteristic but not pathognomonic of a brain abscess. Empiric antimicrobial therapy prior to imaging and a lack of inflammatory response in immunocompromised patients may cause elevated diffusivity. *Bacillus cereus*, tuberculosis, and fungal abscesses may show facilitated diffusion within the abscess cavity and a low signal on DWI [25]. Hemorrhage into the lesion may also prevent the center of an abscess from demonstrating typical high-signal intensity on DWI.

Magnetic resonance spectroscopy is useful to distinguish brain abscesses from brain tumors when hemorrhage is present [26]. Utilization of automated machine learning techniques may improve the differentiation of cerebral metastases from abscesses on MRI [27]. Classification of cerebral ring-enhancing lesions into abscess and glioblastoma using a machine-learning approach has been proposed [28]. Deep learning frameworks for identifying brain abscesses in medical imaging demonstrate good reliability and accuracy, with multi-class categorization under development [29]. Such approaches may enhance decision-making and improve patient outcomes [30].

Periodontitis involves bacterial dysbiosis, dental plaque formation, tissue destruction, and tooth loss [31]. Approximately 10% of the global population is affected by severe periodontitis [32]. There are five routes of odontogenic infections into the brain: (a) systemic hematogenous bacteremia; (b) direct venous drainage; (c) contiguous extension; (d) introduction of foreign bodies; and (e) lymphatic drainage [9]. Upper molars are most often associated with brain abscesses. Multiple abscesses occur in 4.4% of cases. The highest prevalence of involvement is the frontal lobe (25%), followed by temporal, parietal, and occipital lobes (16.2%, 8.8%, and 11.8%, respectively) [33]. The side of the abscess may not coincide with the side of the odontogenic infection [9,33]. This discordance, as illustrated in our case, suggests a hematogenous route of dissemination [33].

There are more than 700 species of bacteria in the oral cavity [34]. The three bacteria most commonly associated with odontogenic brain abscesses are *Streptococcus intermedius*, *Fusobacterium nucleatum*, and *Streptococcus viridans *[33]. Diagnosis of an odontogenic brain abscess is challenging. Across studies, microorganisms were collected and identified from both the brain and the oral cavity in only 17% of cases and from the brain alone in 65% of cases [31]. Anaerobes are mainly prevalent in periodontal pockets [35]. Oral pathologic conditions do not always exhibit clinical signs or symptoms [19].

The three diagnostic criteria for odontogenic brain abscess proposed by Rae Yoo et al. were applicable to our case [8,36,37]: (a) no alternative source of bacteremia; (b) microbiological findings reveal pathogens typically found in oral microflora; and (c) clinical or radiographic signs of active periodontal disease. Our patient presented with clinical and radiological evidence of severe periodontitis. Culture of the drained fluid revealed *E. lenta *and *B. wadsworthia*, aligning with these diagnostic criteria.

*E. lenta*, formerly classified as *Eubacterium lentum *until 1999, is an obligate anaerobic, non-motile, non-sporulating, Gram-positive bacillus and is part of the normal colonic microbiome [38,39]. *E. lenta *has also been isolated in the female genital tract and oral cavity [40]. It is associated with a spectrum of disease, ranging from asymptomatic bacteremia and intra-abdominal infection to disseminated disease [41]. *E. lenta *brain abscess in an immunocompetent adult, associated with cholecystitis leading to multiple abscesses, has been reported [42]. Clinicians should be alerted by a positive culture of *E. lenta *for detailed patient evaluation and abdominal imaging. Our case had a history of diverticulosis, though without abdominal symptoms; contrast CT of the abdomen ruled out intra-abdominal infection.

*E. lenta *is generally susceptible to vancomycin, meropenem, and metronidazole. Resistance to penicillin, ceftriaxone, and cefotaxime is frequent [43]. Increased mortality with piperacillin-tazobactam monotherapy has been reported [44]. Monotherapy with piperacillin-tazobactam or ceftriaxone is not recommended [45].

*B. wadsworthia* is a Gram-negative, rod-shaped, asaccharolytic anaerobic bacterium that exhibits strong catalase positivity and hydrogen sulfide production [46]. The gastrointestinal tract is the preferred niche of *B. wadsworthia*, but it can also be found in saliva and the vagina [47]. Brain abscess due to *B. wadsworthia *in immunocompetent hosts, secondary to chronic otitis media, has been reported [48]. *B. wadsworthia* is sensitive to kanamycin and colistin and resistant to vancomycin, and penicillins show weak activity regardless of beta-lactamase status. Good activity has been reported with meropenem, tigecycline, and moxifloxacin [48,49].

In our case, although the gut source was ruled out and oral disease was present, it is challenging to irrefutably prove that the oral cavity was the sole source, given that these organisms can inhabit multiple sites.

A literature search was conducted on PubMed using the terms “brain abscess”, “Eggerthella lenta”, “Eubacterium lentum”, and “Bilophila wadsworthia”. Titles and abstracts of all English-language articles were analyzed without restrictions on publication date. The search yielded five articles, illustrating the rarity of brain abscess secondary to *E. lenta *or *B. wadsworthia *infection [10,48,50-52].

Ceftriaxone exhibits good blood-brain barrier penetration, which is not affected by concomitant steroid use [53,54]. Empiric ceftriaxone plus metronidazole therapy is effective in most cases, even without pathogen confirmation [7]. Ceftriaxone also significantly impacts the intestinal microbiota by reducing bacterial diversity and the number of Gram-negative enteric bacilli [55]. Metronidazole resistance among anaerobes is generally uncommon, although an increasing trend has been observed [56]. No significant differences between aspiration and open excision of brain abscesses have been shown in functional outcome, mortality, or reoperation rate [57]. Currently, there are no guidelines for dental intervention in odontogenic brain abscess treatment [58].

Traditional culture and morphological identification of anaerobes are limited by their fastidious nature, specific growth requirements, extended incubation times, and difficulty in isolation. Molecular techniques, such as 16S rRNA gene sequencing, assist in identification, particularly in culture-negative specimens, but they do not detect fungi, viruses, or parasites and have limited resolution in distinguishing closely related species or strains [59]. mNGS provides more comprehensive analyses of microbial characteristics and can detect novel organisms, particularly anaerobes [60].

Limitations of mNGS include environmental contamination, incomplete databases for rare anaerobes, background noise from human DNA or reagents, and difficulty distinguishing closely related species. Discrepancies between mNGS and traditional culture results should be carefully interpreted. Conventional anaerobic culture remains essential [61].

## Conclusions

To our knowledge, this is the first report of a brain abscess associated with odontogenic *E. lenta *and *B. wadsworthia *coinfection, highlighting the importance of thorough diagnostic workup and expanding the microbiological spectrum of odontogenic brain abscesses. High awareness of atypical presentations, timely diagnosis, surgical excision, and appropriate empiric antibiotic therapy led to a favorable clinical outcome. The microbiology of odontogenic brain abscesses requires further research, and structured strategies for dental intervention may be beneficial in such cases. Our report emphasizes the need for dental evaluation in patients with brain abscesses. Empiric treatment with ceftriaxone and metronidazole was supported by culture sensitivities, confirming its appropriateness for odontogenic infections. Clinicians should recognize that atypical, stroke-like presentations without systemic signs of infection may occur and require a high index of suspicion.
